# Association Between Hospital Efficiency and Quality of Care Among Fee-for-Service Medicare Beneficiaries with Prostate Cancer: A Retrospective Cohort Study

**DOI:** 10.3390/cancers16244154

**Published:** 2024-12-13

**Authors:** Sumedha Chhatre, S. Bruce Malkowicz, Neha Vapiwala, Thomas J. Guzzo, Ravishankar Jayadevappa

**Affiliations:** 1 Department of Psychiatry, Perelman School of Medicine, University of Pennsylvania, Philadelphia, PA 19104, USA; rasu@pennmedicine.upenn.edu; 2 Leonard Davis Institute of Health Economics, University of Pennsylvania, Philadelphia, PA 19104, USA; 3 Corporal Michael J. Crescenz VAMC, Philadelphia, PA 19104, USA; 4 Abramson Cancer Center, University of Pennsylvania, Philadelphia, PA 19104, USA; 5 Division of Urology, Department of Surgery, Perelman School of Medicine, University of Pennsylvania, Philadelphia, PA 19104, USA; 6 Radiation Oncology, Perelman School of Medicine, University of Pennsylvania, Philadelphia, PA 19104, USA; 7 Department of Medicine, Perelman School of Medicine, University of Pennsylvania, 224 Ralston-Penn Center, Philadelphia, PA 19104, USA

**Keywords:** prostate cancer, hospital technical efficiency, health service use, mortality, racial disparity

## Abstract

Background: Technical efficiency evaluates a hospital’s economic performance and plays an important role in variations in quality of care and outcomes. The study objective was to examine the association between hospital efficiency and quality of care among fee-for-service Medicare beneficiaries with prostate cancer and to assess if race moderates this association. Design: Retrospective study using Surveillance, Epidemiological, and End Results—Medicare (SEER-Medicare) data from 1998 to 2016 for prostate cancer patients aged ≥ 66. We computed hospital technical efficiency using a data envelopment analysis. Outcomes were emergency room visits, hospitalizations, cost, and mortality (all-cause and prostate cancer-specific). We used competing risk regression for survival, log-link GLM models for cost, and Poisson models for count data. The propensity score approach was used to minimize selection bias. Results: The cohort consisted of 323,325 patients. Compared to higher efficiency hospitals (upper quartile), low hospital efficiency (i.e., lower quartile) was associated with a higher hazard of long-term mortality (Hazard ratio (HR) = 1.06, 95% CI = 1.05, 1.08) and long-term prostate cancer-specific mortality (HR = 1.14, 95% CI = 1.11, 1.17). Compared to higher efficiency levels, lower levels were associated with impaired emergency room visits, hospitalizations, and costs. A one unit increase in the efficiency score was associated with greater benefits for cost and mortality for African American and white patients. The benefit was larger for African American patients compared to white patients. Conclusions: Increasing hospital efficiency may help improve outcomes among Medicare prostate cancer patients. Policies to redirect patients to hospitals with higher efficiency can enhance the quality of care and outcomes.

## Simple Summary

Hospitals are an important sector of the U.S. economy. Hospital characteristics such as efficiency play an important role in the quality and cost of care. The impact of differences in hospital efficiency on quality can go either way: efficiency forces hospitals to cut prices and quality or to improve quality. Race and ethnicity may affect the association between hospital efficiency and outcomes. In this study, we examined the association between hospital efficiency and quality of care in fee-for-service Medicare beneficiaries with prostate cancer. We also assessed if race moderates the observed association. We used Surveillance, Epidemiological, and End Results—Medicare (SEER-Medicare) data from 1998 to 2016 for prostate cancer patients aged 66 or older. SEER-Medicare data were linked to American Hospital Association data. We concluded that increasing hospital efficiency may help improve outcomes of prostate cancer care in Medicare fee-for-service patients and provide larger benefits to African American patients compared to white patients. Policies to redirect patients to hospitals with higher efficiency can thus improve quality of care and outcomes.

## 1. Introduction

Hospitals are an important sector of the U.S. economy and the keystone of the healthcare system. Hospital characteristics such as efficiency, competition, revenue, and volume play important roles in treatment, health service use, and quality and cost of care [1]. The association between hospital volume, treatment, quality of prostate cancer care, and other outcomes has been explored [2,3]. Hospital technical efficiency affects the quality of care and outcomes in many ways [4,5,6]. The impact of differences in hospital efficiency on quality can go either way: efficiency forces hospitals to cut prices or improve quality. At the same time, we know very little about the association between efficiency and the racial and ethnic disparity in quality of care. This issue demands further research to aid policymakers in developing market-based strategies to reduce racial and ethnic disparity in the quality of cancer care.

A hospital’s technical efficiency evaluates its economic performance. It measures the proportion of input (e.g., capital and labor) to output (e.g., inpatient days). Although the U.S. healthcare system is recognized for inefficiency, no dramatic breakthroughs have led to more efficient alternatives [4,6,7,8]. Hospital technical efficiency means the same output level can be produced with less input [7,9,10,11]. When units are compared, efficient units produce the same output with fewer inputs than inefficient units or more output with the same input as inefficient ones. Hospital administrators can use hospital efficiency measures to benchmark and focus on hospitals with exceptionally low or high scores. Payers or insurance providers can use efficiency scores to re-appraise payment policy.

In a study of urban U.S. hospitals in 34 states that were operating in 2004, trade-offs between quality and efficiency were assessed. The results indicated that the reallocation of resources may improve the relative quality. Additionally, quality improvement in some dimensions of care need not be due to higher costs or reduced access to care [1]. One study of urban U.S. hospitals in 20 states operating in 2001 assessed the impact of employing a variety of controls for hospital quality and patient burden of illness on the mean estimated inefficiency and relative ranking of hospitals. It was concluded that outcome measures of quality can provide useful insight into a hospital’s operations [8]. A study of German hospitals studied association between ownership status and hospital efficiency. It was observed that private ownership does not necessarily lead to higher efficiency [12]. Studies have reported different conclusions regarding association between efficiency and competition. For example, one study examined the effect of early-stage market competition on later-stage hospital efficiency in Taiwan using data for 102 teaching hospitals for the period between 1996 and 2001. The results indicated a lack of association between market competition and hospital efficiency [5]. On the other hand, using data from public hospitals in the state of Victoria, Australia, researchers observed a positive relationship between efficiency and competition (measured by the Hirschman–Herfindahl Index (HHI)). However, this relationship was negative when the number of competing private hospitals was used instead of HHI [6]. Our comprehensive literature search on efficiency and quality of care in the context of oncology yielded one publication related to colorectal cancer. The study assessed the practice variation in preoperative care between Dutch hospitals in terms of technical efficiency and identified associated factors [13]. Additionally, we identified recent research that has assessed the factors associated with hospital efficiency [14,15,16,17]. One study analyzed the association between hospital completion and hospital efficiency [18].

Hospitals account for a significant proportion of healthcare expenditures. As healthcare reform continues, hospitals will bear a considerable burden to improve their efficiency. The impact of any changes in the efficiency of U.S. hospitals on the quality of their care remains under increasing scrutiny. Hospitals are an important economic enterprise, accounting for approximately 6% of the U.S. GDP; therefore, measuring their financial performance is key to improving resource management [1]. Efficiency means the maximum use of resources to generate returns. Different methods have been applied to measure hospital efficiency, the data envelopment analysis (DEA) and stochastic frontier analysis (SFA) being the most common [19,20]. The frontier-based methods compare hospitals’ actual performance against an estimated efficient frontier. Prior studies have reported that the hospital efficiency scores estimated that using the SFA and DEA are comparable [21,22,23]. A nonparametric method, the DEA is well suited for hospital technical efficiency measurement if a hospital (all types) has multiple inputs and outputs and if the inputs’ and outputs’ market prices are unavailable or difficult to estimate [24,25,26,27,28,29].

In this study, we used the DEA to compute hospital efficiency. The DEA may provide valuable and objective information regarding hospital technical efficiency, resource management, and outcomes [1,4,5,6,30,31]. Considering the importance of hospital performance measurement, this study aims to measure hospital efficiency using the DEA technique.

Prostate cancer ranks as the second leading cause of death among men in the U.S. and is an expensive malignancy to treat. It is expected that in the year 2024, there will be 299,010 new prostate cancer cases and 35,250 deaths related to prostate cancer [32]. Annual expenses for prostate cancer care are estimated to be more than USD 20 billion [33]. A recent nationwide, population-based, retrospective cohort study used SEER-Medicare databases to assess the costs of prostate cancer among men aged 70 years or older. Within 3 years after a prostate cancer diagnosis, the median cost (per patient) was USD 14,453 (interquartile range [IQR], USD 4887–27,899). Costs associated with treatment comprised the majority of this cost (median, USD 10,558; IQR, USD 1990–23,718) [34]. African American men are 1.5 times more likely to be diagnosed and 2.2 times more likely to die of prostate cancer compared with their white counterparts [35,36]. Disparities exist in the quality of care across regions, hospitals, ages, and racial and ethnic groups [2,37,38,39,40,41,42,43,44]. Measuring and monitoring the economic efficiency of prostate cancer care is essential for reducing the spiraling costs and racial disparities in care. In this study, we first use prostate cancer care as a model to analyze the association between hospital efficiency and outcomes (quality of care and cost of care). Quality of care is operationalized as the number of emergency room (ER) visits, number of hospitalizations, all-cause mortality, and prostate cancer-specific mortality. Next, we assess if race (African American and white) modifies the association between efficiency and outcomes. We hypothesize that hospital efficiency is directly associated with the quality of prostate cancer care and inversely associated with costs of care and will benefit both African American and white patients with prostate cancer.

## 2. Materials and Methods

### 2.1. Data Source and Sample

In this cohort study, we used retrospective data from Surveillance, Epidemiology, and End Results (SEER)—Medicare linked database for the period between 1998 and 2016. The SEER-Medicare data are provided by the National Cancer Institute and consist of health service claims and clinical information about Medicare enrollees living in SEER areas.

The SEER cancer registry collects cancer-related information and covers 26% of the United States population [45]. For our study, data from several SEER-Medicare files were used, including (a) Patient Entitlement and Diagnosis File (PEDSF). This file consists of data related to SEER registry and Medicare entitlement. (b) Medicare Provider Analysis and Review file (MEDPAR) was also used. This is related to claims data resulting from hospital inpatient and skilled nursing facility stays. (c) In addition, Outpatient Standard Analytic File (outpatient file) was used. This data file consists of claims that are related to hospital outpatient episodes. Finally, (d) Physician/Supplier File (NCH) was used. This data file contains claims associated with physician and other medical services. Our study was approved by the institutional review board. We linked the SEER-Medicare data with data from American Hospital Association (AHA) to determine data related to hospitals.

### 2.2. Study Cohort

We created a cohort of elderly fee-for-service Medicare beneficiaries who were diagnosed with prostate cancer between 1998 and 2011. As claims for the year before prostate cancer diagnosis are needed to determine comorbidity, our cohort included patients who were at least 66 years of age at diagnosis. We defined the two years following the diagnosis of prostate cancer as the treatment phase, and hospital-based technical efficiency was computed over this period (as explained below). Following this period, a period of at least five years was considered as the follow-up phase.

### 2.3. Dependent Variables

Emergency room visits, hospitalizations, all-cause mortality, and prostate cancer-specific mortality were assessed in the treatment phase (short-term outcomes). We also assessed all-cause mortality and prostate cancer-specific mortality over the entire study period (long-term outcomes). We used outpatient claims to extract emergency room visits (those not leading to inpatient hospitalization), as emergency room visits that resulted in hospitalization were identified as hospitalizations. We operationalized cost as reimbursements. Total cost of care included the reimbursements for inpatient hospitalizations, outpatient episodes, and services rendered by the providers. All-cause mortality data were obtained from both SEER and Medicare. SEER only provides the month and year of death; therefore, we used middle of the month as the day of death and to build SEER’s date of death. Medicare day, month, and year of death were used to construct Medicare’s date of death. We coded a patient’s status as deceased if SEER and/or Medicare indicated as such. We censored those patients who were alive as of the last day of the study, i.e., 31 December 2016. We obtained data on prostate cancer-specific mortality from SEER.

### 2.4. Independent Variables

#### Hospital Technical Efficiency

Hospital technical efficiency was the key independent variable of interest in this study. It is the ability to produce the maximum possible output from a given set of inputs. A hospital is technically efficient if a reduction in any input requires an increase in at least one other input or a decrease in at least one output. We used the DEA to calculate the technical efficiency. The DEA is a nonparametric approach based on linear programming (LP methods) to estimate distance function measures of technical efficiency [7,9,10,11,20,46,47]. Distance function techniques and other LP methods have been widely used in healthcare. Consequently, efficiency scores represent the realization of random variables. Thus, it is natural to regress these efficiency measures on policy (control) variables of interest that may influence hospital technical efficiency. The hospital technical efficiency scores are computed for the selected outputs (*Y*) and inputs (*X*) using the following linear programming formula [20]:

$$\begin{array}{c} \mathrm{Maximize}\;{TE}_{o}\;=\;\frac{\sum_{r=1}^{s}Ur\;Yro}{\sum_{i=1}^{m}vi\;xio}\\ \mathrm{Subject}\;\mathrm{to}\;\;\;\;\frac{\sum_{r=1}^{s}Ur\;Yro}{\sum_{i=1}^{m}vi\;xio}\;\;\leq 1\\ U_{r},\;v_{i}\;>\;0\;\mathrm{for}\;\mathrm{all}\;r\;\mathrm{and}\;i \end{array}$$

where *TE*_o_ = technical efficiency score for each hospital in the set of o = 1,…, n facilities;

*U*_r_ = Weights assigned to output “r”;

*Y*r_o_ = the selected output “r” produced by each hospital in the set “o”;

*v*_i_ = weights assigned to input “i”;

*x*_io_ = the selected input “i” used by each hospital in the set “o”.

The hospital efficiency score ranges from zero to one, and a higher score indicates higher hospital efficiency.

We used annual AHA data and Medicare Cost reports for all identified hospitals in our cohort to generate the technical efficiency scores. For the DEA model, appropriate input and output variables are those that represent the operational characteristics of production units. This helps ensure that DEA results can guide strategies to improve operational efficiency. In our study, the operational unit was a hospital. The input and output variables that we selected were the most relevant and thus most appropriate for our study. To compute technical efficiency, the following six separate direct hospital outputs were used: (1) inpatient days, (2) inpatient care (measured as number of discharges adjusted for case-mix by weighting discharges by DRGs), (3) inpatient surgical procedures, (4) long-term care inpatient days, (5) number of outpatient visits, and (6) ambulatory surgical procedures. The following seven input variables were used: (1) number of acute-care hospital beds, weighted by a scope-of-services index; (2) number of long-term hospital beds; (3) physicians; (4) registered nurses and licensed practical nurses; (5) other clinical labor; (6) non-clinical labor; and (7) long-term care labor (all staff measured in full-time equivalents). Number of acute-care hospital beds, weighted by a scope of service index and the number of long-term hospital beds, were included as proxies for capital the hospitals uses.

### 2.5. Covariates

The covariates were socio-demographic characteristics (age, race, marital status, and census tract poverty index), disease severity, comorbidity, and prostate cancer treatment. We used Medicare inpatient, outpatient, and provider claims one year before prostate cancer diagnosis to develop the Charlson comorbidity index [48]. Exclusive categories of treatments were surgery (alone or multimodal), radiation therapy (alone or with chemotherapy and/or hormone therapy), and no treatment/active surveillance/watchful waiting.

### 2.6. Statistical Analysis

We used ANOVA and χ^2^-tests to compare the demographic and clinical characteristics between quartiles of hospital efficiency scores. ANOVA was used for comparing mean age and efficiency scores between quantiles of hospital efficiency scores. The χ^2^-tests were used to compare proportion of categorical variables between quantiles of hospital efficiency scores. The analytical strategy involved specifying models at the individual patient level. The emergency room visits and hospitalizations were count data and were appropriately modeled using Poisson regressions models. The cost data distribution may be skewed and Generalized Linear Models (GLM) with log-link are well suited to model non-normal data. Finally, survival analysis is preferred to assess the association between efficiency and survival, as these models take into account the time to event. We used Cox proportional hazard models to analyze survival. The quartiles of hospital efficiency scores were the main independent variable of interest.

Next, we conducted a sub-analysis for African American and white patients. We operationalized the efficiency score as a continuous variable and assessed the moderating effect of race (African American vs. white) on the association between efficiency score and outcomes. As treatment for prostate cancer is non-random, we used propensity score to address any observed confounders [49]. With the help of multi-nominal logistic regression models, we determined the propensity of receiving a given treatment after controlling covariates such as age, race, marital status, census tract poverty indicator, and Charlson comorbidity score. All analytical models were weighted by the inverse probability of treatment obtained from the propensity score. Statistical Analysis System (SAS), Version 9.4 (SAS Institute, Cary, NC, USA) was used for data analysis.

## 3. Results

**Figure 1:**
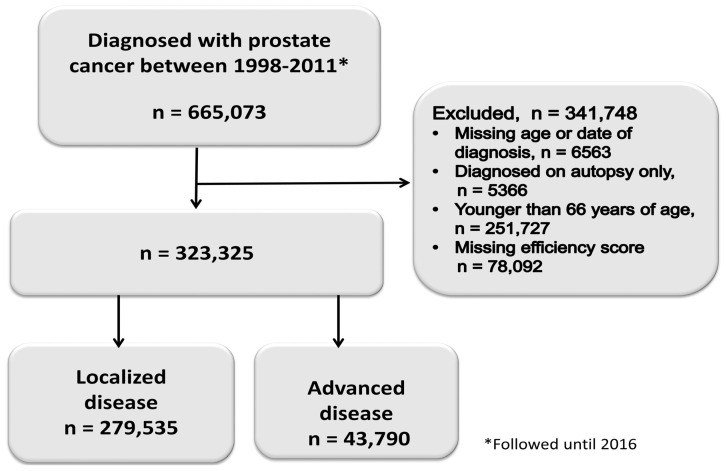
Prostate cancer cohort selection.

**Figure 2:**
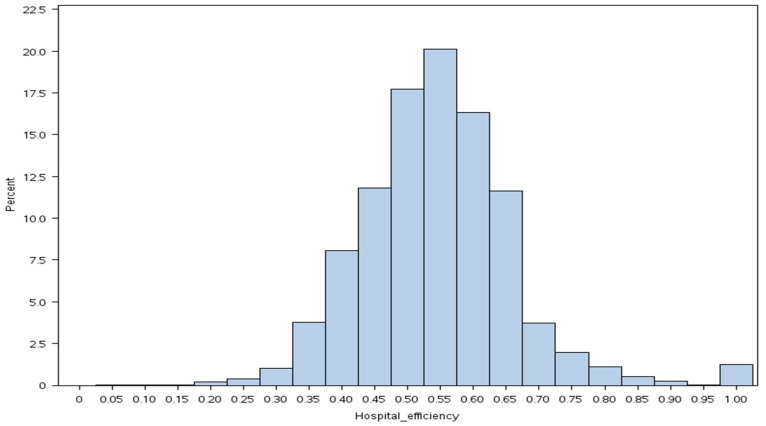
Histogram of hospital technical efficiency scores.

Between 1998 and 2011, there were 665,073 new cases of prostate cancer. After applying our criteria, we retained a cohort of 323,325 prostate cancer patients (Figure 1). Table 1 compares demographic and clinical characteristics by efficiency score quartiles. Figure 2 presents the distribution of hospital-level technical efficiency scores. The distribution appeared to be moderately skewed. The mean technical efficiency score was 0.54 (range 0.15 to 1.00).

An unadjusted comparison of outcomes between quartiles of hospital efficiency scores is reported in Table 2. More than half (51.7%) of the lowest quartile had at least one hospitalization visit in the treatment phase. This proportion was 50.1%, 49.7%, and 50.3% for the 2nd, 3rd, and 4th quartiles, respectively. Similarly, 31.4% of the lowest quartile had at least one ER visit in the treatment phase. This proportion was 29.2%, 28.9%, and 27.3% for the 2nd, 3rd, and 4th quartiles, respectively. Short-term, mean Medicare reimbursement was 24,600 (standard deviation USD 31,196), 23,522 (28,948), 24,087 (30,128), and 23,668 (30,853) for the 1st, 2nd, 3rd, and 4th quartiles of the hospital efficiency score, respectively. Long-term all-cause and prostate cancer-specific mortality was 59.9% and 11.7%, respectively, for the lowest quartile of the efficiency score. These proportions were 59.0% and 11.1%, 58.5% and 11.3%, and 55.6% and 10.9% for the 2nd, 3rd, and 4th quartiles of the hospital efficiency score, respectively.

As reported in Table 3, compared to the highest quartile of the efficiency score, all lower quartiles of the efficiency score had higher ER visits (incidence rate ratio (IRR) = 1.35, 95% confidence interval (CI) = 1.34, 1.36; IRR = 1.23, 95% CI = 1.22, 1.25; and IRR = 1.16, 95% CI = 1.14, 1.17, respectively). Similarly, hospitalizations and cost of care were higher for the lower efficiency score quartiles than the highest quartile. The hazard of long-term all-cause mortality was higher for the three lower quartiles of the hospital efficiency score compared to the highest quartile (Hazard ratio (HR) = 1.06, 95% CI = 1.05, 1.08, HR = 1.06, 95% CI = 1.05, 1.07; and HR = 1.07, 95% CI = 1.05, 1.08, respectively). Similar observations were made for long-term prostate cancer-specific mortality and short-term mortality (all-cause and prostate cancer-specific). The results of the sub-analysis are presented in Table 4.

Emergency Room Visits: As seen from Model 1 (Table 4), the percent change in the incident rate of ER visits associated with a one unit increase in the efficiency score was 64% lower, holding other variables constant. Model 2 showed a statistically significant interaction between race and the efficiency score. For white prostate cancer patients, the percent change in the incident rate of ER visits associated with a one unit increase in the efficiency score was 54% lower (IRR = 0.46, 95% CI = 0.45, 0.49). However, the interaction between the efficiency score and race was insignificant for African Americans (IRR = 1.08, 95% CI = 0.89, 1.28).

Hospitalizations: The main effects of the association between the efficiency score and hospitalizations (IRR, 0.67, 95% CI, 0.65–0.69) were observed from Model 1 (Table 4). Model 2 showed a statistically significant interaction between race and the efficiency score. For white prostate cancer patients, the percent change in the incidence rate of hospitalizations associated with a one unit increase in the efficiency score was 33% lower (IRR = 0.67, 95% CI = 0.64, 0.70). The association of higher efficiency with hospitalization for African American prostate cancer patients was statistically non-significant (IRR = 0.99, 95% CI = 0.91, 1.08).

Cost: The main effects of the association between the efficiency score and cost (e^β^ = 0.73, 95% CI, 0.72–0.75) were observed from Model 1 (Table 4). Model 2 showed a statistically significant interaction between race and the efficiency score. For white prostate cancer patients, the cost reduction associated with a one unit increase in the efficiency score was 35% (e^β^ = 0.65, 95% CI = 0.64, 0.66). The effect of higher efficiency for African American prostate cancer patients was 0.71 times that of their white counterparts (e^β^ = 0.71, 95% CI = 0.70, 0.72). The percent change in costs associated with a one unit increase in the efficiency score was 54% lower for African Americans patients.

All-cause mortality (short-term): As presented in Table 4, the main association between the efficiency score and short-term all-cause mortality (HR, 0.91, 95% CI, 0.87–0.94) was observed from Model 1. As observed from Model 2, there was a significant interaction between race and the efficiency score. Among white prostate cancer patients, a 5% lower hazard of short-term all-cause mortality was associated with a one unit increase in efficiency (HR = 0.95, 95% CI = 0.91, 0.99). For African American patients, the effect of higher efficiency was 0.65 times that experienced by white patients (HR = 0.65, 95% CI = 0.58, 0.72). One unit increase in the efficiency score translated into 38% lower hazard of short-term all-cause mortality for African American patients.

Prostate cancer-specific mortality (short-term): The main effects of the association between efficiency and short-term prostate cancer-specific mortality (HR, 0.70, 95% CI, 0.64–0.76) was observed from Model 1 (Table 4). Model 2 showed a significant interaction between race and the efficiency score. For white prostate cancer patients, the hazard of short-term prostate cancer-specific mortality associated with a one unit increase in the efficiency score was 23% lower (HR = 0.77, 95% CI = 0.70, 0.84). The effect of higher efficiency for African American prostate cancer patients was 0.38 times that of their white counterparts (HR = 0.38, 95% CI = 0.29, 0.47). The percent change in hazard of short-term all-cause mortality associated with a one unit increase in the efficiency score was 70% lower for African Americans.

All-cause mortality (long-term):

As shown in Model 1, Table 4, we observed the main effects of the efficiency score for long-term all-cause mortality (HR, 0.83, 95% CI, 0.80–0.87). A statistically significant interaction between race and the efficiency score was observed from Model 2. White prostate cancer patients had a 13% lower hazard of long-term all-cause mortality for a one unit increase in efficiency (HR = 0.87, 95% CI = 0.83, 0.90). For African American patients, the percent change in the hazard of short-term all-cause mortality was 45% lower for a one unit increase in the efficiency score.

Prostate cancer-specific mortality (long-term): As presented in Model 1, Table 4, the main effects between the efficiency score and long-term prostate cancer-specific mortality were observed (HR, 0.66, 95% CI, 0.60–0.72). Next in Model 2, we noticed a significant interaction between race and the efficiency score. The hazard of long-term prostate cancer-specific mortality associated with a one unit increase in the efficiency score was 29% lower for white patients (HR = 0.71, 95% CI = 0.65, 0.78). Compared to white patients, the effect of higher efficiency for African American prostate cancer patient was 0.39 times (HR = 0.39, 95% CI = 0.31, 0.49). For African American patients, there was a 72% lower hazard of short-term all-cause mortality associated with a one unit increase in the efficiency score.

## 4. Discussion

Our results suggest that higher efficiency was associated with (1) decreased short-term ER visits, hospitalizations, cost, all-cause mortality, and prostate cancer-specific mortality; (2) a decreased hazard of long-term all-cause mortality and prostate cancer-specific mortality; and (3) greater benefits to African American patients in terms of cost, all-cause mortality (both short-term and long-term), and prostate cancer-specific mortality (both short-term and long-term). Prostate cancer is one of the most expensive malignancies and the second leading cause of death among men in the U.S. Monitoring and assessing hospital efficiency and the quality of prostate cancer care is essential for reducing spiraling costs. One Dutch study observed variation in the technical efficiency of preoperative colorectal cancer care for older patients. High volume and the availability of a care pathway, including pre-habilitation, were positively associated with technical efficiency of hospitals [13].

In our study, we used the DEA to compute hospital efficiency. The DEA may provide useful and objective information regarding hospital technical efficiency, resource management, and outcomes [1,4,5,6,30,31]. The healthcare competition theory discusses higher degrees of specialization and focuses on disease-specific services to improve outcomes, competitiveness, and efficiency [4,11]. Research has indicated that the number of competitors in the market contributed positively to technical efficiency. Additionally, the differences in efficiency scores were attributed to environmental factors, including ownership, market structure, and regulation effects [11]. Prior studies have identified that hospital efficiency was directly associated with hospital competition and hospital type [6,31,50,51,52,53,54]. Studies have reported varying conclusions regarding the association between efficiency and competition. One study in Taiwan used data for 102 teaching hospitals for the period between 1996 and 2001. The results showed a lack of association between market competition and hospital efficiency [5]. Using data from public hospitals in the state of Victoria, Australia, a study observed a positive association between efficiency and competition [6].

One of the important indicators of hospital performance is hospital efficiency. Hospitals that were high-efficiency tended to have larger practices [13], lower average costs, higher labor productivity, and higher profit margins [4,51,52] compared to hospitals who were least efficient. Additionally, hospitals that are most efficient are likely to be nonteaching, be investor owned, and belong to multi-hospital systems [20,30,50]. The competition and penetration of health maintenance organizations was lower in the areas where hospitals from the high-efficiency group were located. These hospitals also had a higher share of Medicaid and Medicare admissions [53]. Analyses suggest that public policies can improve outcomes and reduce disparities by improving hospital efficiency [53,54].

Policymakers should recognize that low hospital efficiency is one of the most important problems of the U.S. healthcare system. Higher efficiency was associated with improved outcomes and provided larger benefits to African American patients than white patients. Data envelopment analyses may provide useful and especially objective information regarding the technical efficiency of hospital care and support hospital management and policymakers’ decisions [4]. Appropriate incentive-based policy measures can facilitate improvement in hospital efficiency. One way to increase hospital efficiency is through competition. Also, by eliminating inefficiency, one can improve the level of output using the same level of resources [54]. Future research should focus on the process of care measures such as underuse, overuse, and misuse of care and its association with efficiency and quality of care.

## 5. Conclusions

We first begin with strengths and limitations of our study. To our knowledge, currently, there are no studies that have examined the association between hospital technical efficiency and outcomes among prostate cancer patients. Our study uses population-based, longitudinal data to assess the association between hospital technical efficiency and short-term and long-term outcomes among prostate cancer patients. The use of the well-established DEA technique is another strength of our study. Finally, we address the moderating effects of race on the associations between hospital technical efficiency and outcomes.

We also note some limitations of our study. First, due to the observational nature of our data, we could not establish a causal relationship between hospital efficiency and outcomes of care. Second, some residual bias may exist even after adjusting for the propensity score to minimize selection bias. Third, our study lacks intermediate outcomes, like cancer recurrence or distant disease control. Future studies will address the associations between efficiency and disease progress. However, we did observe an association between hospital efficiency and mortality that calls for further investigation. We excluded Hispanic and other racial and ethnic groups due to the small number. Our study included fee-for-service Medicare beneficiaries who were at least 66 years of age, were not enrolled in an HMO, and were residing in a SEER area. The distribution of age and race and ethnicity for beneficiaries aged 66 years or older is comparable to that of older adults in the U.S. However, the SEER areas report a higher proportion of non-white persons. Finally, mortality rates obtained from SEER may not be representative of U.S. cancer mortality rates [45].

We also note certain limitations of the DEA technique. Since the DEA is an extreme point technique, noise such as measurement error can lead to significant problems. While the DEA is good at estimating the “relative” efficiency of a decision-making unit, its convergence to “absolute” efficiency is slow.

Hospital efficiency is an important indicator of hospital performance. This novel longitudinal study used the DEA to assess hospital efficiency from 1998 to 2016. The results showed that hospital efficiency was associated with quality-of-care measures and cost of care. Additionally, race modified the association between efficiency and cost, all-cause mortality, and prostate cancer-specific mortality. To eliminate mismanagement of resources, it is necessary to improve and monitor the hospital efficiency indicators.

## Figures and Tables

**Table 1:** Unadjusted comparison of outcomes for prostate cancer patients aged 66 and older and diagnosed between 1998 and 2011, stratified by quartile of efficiency score, n = 323,325.

	Efficiency Groups (Quartile Based)				
	Q1 (n = 80,369)	Q2 (n = 80,963)	Q3 (n = 82,340)	Q4 (n = 79,653)	*p*
Age at diagnosis, mean ± std	75.0 (6.2)	74.9 (6.2)	74.8 (6.1)	74.5 (6.1)	<0.0001
Race					<0.0001
White	61,180 (76.1)	63,892 (78.9)	62,586 (76.0)	59,812 (75.1)	
African American	8158 (10.2)	8790 (10.9)	10,743 (13.1)	9011 (11.3)	
Hispanic	6692 (8.3)	5219 (6.5)	5316 (6.5)	5158 (6.5)	
Others	4339 (5.4)	3062 (3.8)	3695 (4.5)	5672 (7.1)	
Marital status					<0.0001
Married	53,128 (66.1)	54,016 (66.7)	53,875 (65.4)	53,403 (67.0)	
Census tract poverty indicator					<.0001
0% to <5%	18,899 (23.5)	22,358 (27.6)	23,913 (29.0)	27,291 (34.3)	
5% to <10%	22,019 (27.4)	23,238 (28.7)	22,211 (26.9)	21,312 (26.8)	
10% to <20%	23,149 (28.8)	21,267 (26.3)	21,045 (25.6)	19,188 (24.1)	
20% to 100%	15,854 (19.7)	13,758 (16.9)	14,821(18.0)	11,584 (14.5)	
Unknown	448 (0.56)	342 (0.42)	350 (0.43)	278 (0.35)	
Comorbidity					<0.0001
0	42,383 (52.7)	42,623 (52.7)	42,690 (51.9)	40,348 (50.7)	
1	33,498 (41.7)	34,090 (42.1)	35,136 (42.7)	35,320 (44.3)	
≥2	4488 (5.6)	4250 (5.3)	4514 (5.5)	3985 (5.0)	
Grade					<0.0001
Well-differentiated	2026 (2.5)	1699 (2.1)	1782 (2.2)	1452 (1.8)	
Moderate-diff	38,949 (48.5)	39,522 (48.8)	40,459 (49.1)	37,777 (47.4)	
Poor/undifferentiated	33,861 (42.1)	34,450 (42.6)	34,636 (42.1)	35,156 (44.1)	
Unknown	5533 (6.7)	5292 (6.5)	5462 (6.6)	5268 (6.6)	
Stage					<0.0001
Local	69,171 (86.1)	70,293 (86.8)	71,371 (86.7)	68,700 (86.3)	
Advanced	11,198 (13.9)	10,670 (13.2)	10,969 (13.3)	10,953 (13.8)	
Treatment					<0.0001
Surgery (alone/with radiation and/or chemo)	17,001 (21.2)	16,457 (20.3)	16,430 (19.9)	17,572 (22.1)	
Radiation (alone or with chemo)	40,204 (50.0)	40,461 (49.9)	42,616 (49.9)	40,026 (50.2)	
None	23,164 (28.8)	24,045 (29.7)	24,045 (29.7)	22,055 (27.7)	
Hospital Efficiency score mean ± standard deviation	0.41 ± 0.05	0.51 ± 0.02	0.57 ± 0.02	0.69 ± 0.10	<0.0001

**Table 2:** Unadjusted comparison of outcomes for prostate cancer patients aged 66 and older and diagnosed between 1998 and 2011, stratified by level of quartile of hospital efficiency score, n = 323,325.

Outcome	Q1 (n = 80,369)	Q2 (n = 80,963)	Q3 (n = 82,340)	Q4 (n = 79,653)	*p* Value
Short-term (treatment phase, i.e., within two years post prostate cancer diagnosis) outcomes					
Health service use, n (%)					
Number of hospitalizations					<0.0001
0	38,513 (47.9)	40,477 (49.9)	41,412 (50.3)	39,665 (49.8)	
1	22,296 (27.4)	22,551 (27.9)	22,715 (27.6)	23,145 (29.1)	
≥2	19,560 (24.3)	17,935 (22.2)	18,213 (22.1)	16,843 (21.2)	
Number of ER visits					<0.0001
0	55,136 (68.6)	57,349 (70.8)	58,557 (71.1)	57,899 (72.7)	
1	9653 (12.1)	9198 (11.4)	9500 (11.4)	8918 (11.2)	
≥2	15,580 (19.4)	14,416 (17.8)	14,283 (17.4)	12,836 (16.1)	
All-cause	9188 (11.4)	8620 (10.7)	9074 (11.0)	8116 (10.2)	<0.0001
Prostate cancer-specific	3070 (3.8)	2737 (3.4)	3021 (3.7)	2636 (3.1)	<0.0001
Total Medicare reimbursement in USD,					<0.0001
Mean ± standard deviation	24,600 ± 31,196	23,522 ± 28,948	24,087 ± 30,128	23,668 ± 30,853	
Long-term (up to 18 years) mortality, n (%)					
All-cause	48,203 (59.9)	47,781 (59.0)	48,136 (58.5)	44,260 (55.6)	<0.0001
Prostate cancer-specific	9427 (11.7)	8999 (11.1)	9318 (11.3)	8650 (10.9)	<0.0001

**Table 3:** Association between quartile of hospital efficiency score and outcomes for prostate cancer patients aged 66 and older, diagnosed between 1998 and 2011, weighted by propensity score *, n = 323,325.

Efficiency Score Quartile	Emergency Room Visit IRR ** (95% CI)	Hospitalizations IRR ** (95% CI)	Medicare Reimbursement e^β^ *** (95% CI)	Short-Term All-Cause Mortality HR ^&^ (95% CI)	Short-Term Prostate Cancer Specific Mortality HR ^&^ (95% CI)	Long-Term All-Cause Mortality HR ^&^ (95% CI)	Long-Term Prostate Cancer Specific Mortality HR ^&^ (95% CI)
Q1	1.35 (1.34, 1.36)	1.15 (1.14, 1.17)	1.08 (1.07, 1.10)	1.04 (1.03, 1.05)	1.13 (1.09, 1.16)	1.06 (1.05, 1.08)	1.14 (1.11, 1.17)
Q2	1.23 (1.22, 1.25)	1.10 (1.08, 1.11)	1.03 (1.01, 1.04)	1.04 (1.02, 1.05)	1.03 (1.00, 1.06)	1.06 (1.05, 1.07)	1.04 (1.01, 1.07)
Q3	1.16 (1.14, 1.17)	1.11 (1.09, 1.12)	1.04 (1.02, 1.06)	1.03 (1.02, 1.04)	1.07 (1.05, 1.10)	1.07 (1.05, 1.08)	1.09 (1.06, 1.12)
Q4 (Ref)	-	-	-			-	-

* All models were also adjusted for age, marital status, Charlson comorbidity score, grade, and treatment. ** IRR = Incidence rate ratio. *** e^β^ = Exponent of beta estimate. ^&^ HR = Hazard ratio.

**Table 4:** Summary of two series of models on the interactive effects of race and hospital efficiency score on outcomes for prostate cancer patients age 66 and older, diagnosed between 1998 and 2011, weighted by propensity score *, n = 323,325.

	Model 1: Main Effects	Model 2: Interaction
Short-term (treatment phase, i.e., within two-years post prostate cancer diagnosis) outcomes		
**ER visit**	IRR (95% CI) **	IRR (95% CI) **
Race (African American)	1.15 (1.14, 1.17)	
Efficiency score	0.36 (0.35, 0.37)	
Efficiency score × White		0.46 (0.45, 0.49)
Efficiency score × African American		1.08 (0.89, 1.28)
**Hospitalization**	IRR (95% CI) **	IRR (95% CI) **
Race (African American)	1.12 (1.11, 1.13)	
Efficiency score	0.67 (0.65, 0.69)	
Efficiency score × White		0.67 (0.64, 0.70)
Efficiency score × African American		0.99 (0.91, 1.08)
**Direct Medical Care Cost**	e^β^ (95% CI) ***	e^β^ (95% CI) ***
Race (African American)	1.03 (0.99, 1.04)	
Efficiency score	0.73 (0.72, 0.75)	
Efficiency score × White		0.65 (0.64, 0.66)
Efficiency score × African American		0.71 (0.70, 0.72)
**All-cause Mortality**	**HR (95% CI) ^&^**	**HR (95% CI) ^&^**
Race (African American)	1.07 (1.05, 1.09)	
Efficiency score	0.91 (0.87, 0.94)	
Efficiency score × White		0.95 (0.91, 0.99)
Efficiency score × African American		0.65 (0.58, 0.72)
**Prostate Cancer-specific Mortality**	**HR (95% CI) ^&^**	**HR (95% CI) ^&^**
Race (African American)	1.21 (1.17, 1.24)	
Efficiency score	0.70 (0.64, 0.76)	
Efficiency score × White		0.77 (0.70, 0.84)
Efficiency score × African American		0.38 (0.29, 0.47)
Long-term (up to 18 years) mortality		
**All-cause Mortality**	**HR (95% CI) ^&^**	**HR (95% CI) ^&^**
Race (African American)	1.04 (1.03, 1.06)	
Efficiency score	0.83 (0.80, 0.87)	
Efficiency score × White		0.87 (0.83, 0.90)
Efficiency score × African American		0.63 (0.56, 0.70)
**Prostate Cancer-specific Mortality**	**HR (95% CI) ^&^**	**HR (95% CI) ^&^**
Race (African American)	1.19 (1.15, 1.22)	
Efficiency score	0.66 (0.60, 0.72)	
Efficiency score × White		0.71 (0.65, 0.78)
Efficiency score × African American		0.39 (0.31, 0.49)

* All models were also adjusted for age, marital status, Charlson comorbidity score, grade, and treatment. ** IRR = Incidence rate ratio. *** e^β^ = Exponent of beta estimate. ^&^ HR = Hazard ratio.

## Data Availability

The data that support the findings of this study are available from SEER-Medicare. Restrictions apply to the availability of these data, which were used under license for this study.
